# The Spanish REAL project: expert allergist guidance on allergen immunotherapy based on mixtures from different allergenic sources

**DOI:** 10.3389/falgy.2026.1736462

**Published:** 2026-01-30

**Authors:** M. Viñas, V. Bellido, D. El-Qutob, F. García, T. W. Jimenez-Rodriguez, M. Rial, J. M. Vega-Chicote, J. Cuesta-Herranz

**Affiliations:** 1 Allergy Department, Consorci Sanitari de Terrassa, Hospital Universitari de Terrassa, Barcelona, Spain; 2 Allergy Department, Hospital El Tomillar, Alcalá de Guadaira, Sevilla, Spain; 3 Internal Medicine Department, Hospital Universitario de La Plana de Vila-real, Castellón, Spain; 4 Allergy Department, Instituto Investigacion Sanitaria Biobizkaia, Hospital Universitario de Basurto, Bilbao, Spain; 5 Allergy Section, Dr. Balmis General University Hospital; Alicante Institute for Health and Biomedical Research (ISABIAL), Alicante, Spain; 6 Allergy Department, Complexo Hospitalario Universitario de Ferrol, Ferrol (A Coruña), Spain; 7 Allergy Department, Hospital Regional Universitario de Málaga, Málaga, Spain; 8 Allergy Department, Instituto de Investigación Sanitaria Hospital Universitario Fundación Jiménez Díaz (IIS-FJD, UAM), Madrid, Spain

**Keywords:** allergen immunotherapy (AIT), immunotherapy mixtures, polyallergy, polymerized extract, polysensitization, polysensitized

## Abstract

**Background:**

The increase in polyallergy highlights allergen-specific immunotherapy (AIT) as a crucial therapeutic option for these patients. It is the only etiological treatment capable of modifying the natural course of allergic diseases. Its use, particularly with mixtures from different allergenic sources, varies across regions. In Spain, geographic and environmental diversity leads to complex, heterogeneous sensitization patterns not fully addressed by international guidelines, reinforcing the need for context-specific recommendations integrating current evidence and real clinical practice.

**Objective:**

To establish general recommendations for managing polyallergic patients undergoing AIT with mixtures of different allergenic sources in Spain.

**Methodology:**

Eight regional meetings with 61 experts were held across Spain using the Workmat® methodology: evidence-based exercises designed to facilitate discussion and consistent data collection.

**Results:**

Experts agreed that polymerized allergen extract mixtures offer several advantages over AIT with simultaneous use of individual allergen extracts, as they allow multiple sensitizations to be treated in a single application, simplifying the treatment without compromising safety or efficacy while also saving time and costs and improving treatment adherence. Furthermore, the use of polymerized allergen extracts has improved efficacy and safety in AIT. It is essential to perform an individualized evaluation, according to the type of extract, the mixture, and the patient's comorbidities.

**Conclusion:**

The increasing prevalence of polyallergy has emerged as a growing challenge in routine clinical practice. In this context, AIT using polymerized allergen extract mixtures, at optimal doses, tailored to individual sensitization profiles represent a practical and effective approach for managing polyallergy in the Spanish clinical setting.

## 1 Introduction

Allergic diseases represent a significant global health concern because of their high prevalence and substantial impact on morbidity and quality of life (QoL) (1), with an increasing number of individuals affected by several allergic conditions (2). Sensitization to allergens is an important pathogenic risk factor in the development of diseases such as allergic rhinitis (3).

A significant complexity in managing allergic disease stems from the high prevalence of polysensitization, where most respiratory allergy patients are sensitized to multiple allergen sources simultaneously (4). As a result, many of these patients may also be clinically polyallergic, exhibiting symptomatic responses to two or more allergenic sources (5). Depending on the population studied, the prevalence of polysensitization ranges widely, from 27.5% to 97.4% (6), with estimates indicating that around 70% of allergic patients attending clinics in Spain in 2015 were polysensitized (7). For instance, the ODISSEE study revealed that 62% of 4,227 allergic patients assessed by 264 physicians were polysensitized (8). Moreover, Llaguno et al. conducted a sensitization profile study including 450 allergic patients from different regions of Spain. Overall, 79% of patients showed sensitization to two or more allergenic sources (9).

Polysensitization has been associated with increased disease severity and higher risk of developing asthma in patients with allergic rhinitis (5). In fact, a US study of 1,338 patients with a medical diagnosis of mild-to-moderate asthma, showed that up to 81% of the sensitized patients reacted to three or more allergenic sources (10). Polyallergy is defined elsewhere as a known causal link between exposure to two or more individual sensitizing allergens and the subsequent development of pertinent clinical symptoms of allergy (5).

Allergen-specific immunotherapy (AIT) remains the only etiological treatment option capable of modifying the natural course of the disease in polysensitized patients with allergic respiratory diseases, directly targeting the underlying pro-inflammatory immune response, thereby reducing both allergic symptoms and the need for symptomatic medication (11–17). In addition, it has the potential to decrease sensitization to additional allergens (18), and reduce the risk of progression from allergic rhinitis to asthma (19–21).

Several real-world observational studies have demonstrated the effectiveness and safety of AIT in both polysensitized and polyallergic patients (5, 22). However, there is no agreement on the therapeutic approach to polysensitized subjects, and the European and US practice guidelines for AIT differ significantly (23, 24). Most AIT prescriptions in the US include multi-allergen mixtures with an average of eight different compounds in the same vaccine, whereas only 20% to 40% of immunotherapies in Europe include multi-allergen formulations (8, 25) A polysensitized patient is not necessarily polyallergic, therefore numerous sensitizations may not always represent a clinical issue, depending on the seasonal pattern of multiple allergen exposure (8, 25) In this context, the European Medicine Agency (EMA) encourages allergists to minimize the combination of unrelated allergenic sources and to avoid combining seasonal and perennial allergens, or allergens with proteolytic activity, unless justified (19).

However, the applicability of this approach may be limited in certain national contexts where allergenic exposure is especially complex, and AIT with allergen mixtures targeting multiple allergies simultaneously could enhance patient adherence and quality of life (6).

In Spain, the clinical management of polyallergic patients presents an additional challenge due to the country's marked geographic and environmental diversity. Regional variability in allergen exposure—shaped by distinct climates, vegetation, and pollution levels—gives rise to heterogeneous sensitization profiles across the territory. In this regard, Martínez-Cañavate et al. described heterogeneous patterns of aeroallergen sensitization in children across different regions of Spain, influenced by environmental factors, climate conditions, and urbanization levels (26). These differences can significantly affect both clinical manifestations and therapeutic decisions, underscoring the need for tailored guidance that reflects the Spanish setting (26).

Recent molecular mapping of allergic sensitization in Europe further reinforces this need for region-specific guidance (27). This high-resolution atlas of exposome- and climate-dependent IgE reactivity demonstrated substantial regional variability across Europe and even within Spain (27). The identification of strongly varying molecular IgE profiles in geographically close areas underscores the importance of adapting allergen-specific immunotherapy prescriptions to local exposure conditions and supports the development of tailored recommendations for the Spanish context.

Despite the rising prevalence of polyallergic patients, the absence of standardized and practical clinical guidelines continues to result in significant variability in management approaches. This issue is especially pronounced when determining the indications, composition, and administration strategies for multi-allergen AIT formulations.

In this context, the REAL Project (REimagining the management of polyALlergy through evidence-based debate) was specifically designed to reflect these unique allergenic exposure patterns across various regions of Spain. The project aimed to establish expert-based recommendations for the appropriate use of immunotherapy mixtures containing allergens from different sources in polyallergic patients. Through structured discussions using the Workmat® methodology, 61 expert allergists explored current evidence and real-world experience to reach practical recommendations.

This initiative also sought to define criteria for identifying patients who may benefit from AIT based on different allergenic sources and to highlight the key factors influencing prescription decisions. By focusing on the Spanish context, the REAL Project addresses the limitations of general European guidelines and provides localized, practical recommendations aligned with the realities of national clinical practice.

## 2 Methods

### 2.1 Participants and project design

The REAL project was designed with a qualitative methodology to explore the perspectives of allergology experts across Spain. The recommendations emerged from discussions that aimed to align current scientific evidence with the practical realities faced in daily clinical practice in order to define recommendations in the management of polyallergic patients using AIT based on mixtures from different allergenic sources.

The scientific committee was composed of eight expert allergists with extensive experience in the diagnosis and management of polyallergy. Each member represented a Spanish region with distinct allergenic exposure patterns, ensuring that the project reflected the country's diverse sensitization profiles (See Supplementary Figure S1). The regions included:

Region 1: Cataluña and Islas Baleares
Region 2: Castellón and Valencia
Region 3: Alicante and Murcia
Region 4: Madrid, Castilla y León and Castilla-La Mancha
Region 5: La Coruña (Galicia)
Region 6: Bilbao, La Rioja, Navarra and Cantabria
Region 7: Málaga (Eastern Andalucía)
Region 8: Sevilla and Huelva (Western Andalucía)

The scientific committee members led the initiative and recruited participants for each regional meeting, with each session moderated by the corresponding committee member. Meetings were conducted in person and were not anonymous, allowing open discussion among experts. The discussion materials used in the workshops were developed by an independent health-care consultancy with methodological expertise, ensuring sponsor independence. The sponsor's role was limited to funding and logistical support.

The first meeting with the scientific committee, held in September 2024, focused on identifying the key clinical aspects to consider for the use of AIT based on allergen mixtures from different allergenic sources, and focusing on how, when, and why such treatment should be used. The main benefits and barriers of multi-allergen immunotherapy were also discussed. The eight regional meetings were held between November and December 2024 following the Workmat® methodology. Each meeting was attended by 6–9 experts and was chaired by the scientific committee member representing the corresponding region. After every session, a report summarizing key outcomes and controversies was created. In January 2025, the scientific committee met to compare regional results, assess differences and similarities between the selected geographical areas, explore their causes and consolidate findings. Drawing on their in-depth understanding of the regional data, the committee members identified general trends and validated a set of recommendations applicable at the national level. No major discrepancies were observed between regions; minor variations reflected local clinical practice and the realities of managing polyallergic patients, which are discussed in the manuscript.

### 2.2 The workmat® methodology

The Workmat® methodology is a structured, evidence-based approach designed to promote collaborative debate and the exchange of expert clinical perspective (28–30). It was implemented in the 8 regional meetings and enabled systematic and equitable data collection across groups.

During each session, experts worked in small groups to answer targeted questions and complete related exercises displayed on posters. The original materials used during the workshops are included in the Supplementary Material to allow readers to understand the structure of the exercises and the topics addressed in each session. Likert-type scales were used for scoring, ranging from 1 to 9, being 1 to 3 (not important/disagreement), 4 to 6 (neutral), and 7 to 9 (important/agreement). The percentage of experts scoring within each of these ranges was calculated to assess the level of agreement among experts to support the recommendation.

Each regional meeting followed a three-phase structure:

**Presentation of evidence:** The regional scientific committee member reviewed the most relevant data.
**Group work:** Experts completed the Workmat® exercises in small groups, engaging in discussion to reach internal consensus.
**Discussion and scoring:** Each group presented its conclusions. An inter-group discussion was then held to consolidate findings and agree on a regional consensus, with scores averaged across groups.

At the national level, the scientific committee consolidated all regional outcomes. This process served as internal validation, integrating perspectives across regions to produce robust recommendations. Final analysis included means, medians, standard deviations, and ranges based on regional scores. Results were visually represented using bar charts, and spider maps were generated in each region to illustrate median scores related to AIT administration patterns. Detailed regional-level agreement data are provided in the Supplementary Material.

## 3 Results

### 3.1 Participants profile

A total of 61 experts were invited to participate in the regional meetings. Demographic characteristics and clinical experience of participants can be found in Table 1. Experts were predominantly female (77.78%), working mostly in public centers (84.44%), and having more than 10 years of experience in the management of AIT (64.44%). Out of the regions represented on the panel, region 4 (Comunidad de Madrid, Castilla y León and Castilla-La Mancha) had the highest representation (*n* = 9; 20.00%), followed by region 6 (Bilbao, La Rioja, Navarra and Cantabria) (*n* = 7; 15.55%).

**Table 1:** Characteristics of included panelists.

Participants^a^/Invited (%)	45/61 (73.77%)
Gender, *n* (%)	
Male	10 (77.78%)
Female	35 (22.22%)
Age (in years), mean (±SD)	47.69 (±10.06)
Professional activity, %	
Public	84.44%
Private	13.33%
Publicly funded private facility	2.22%
Years of experience as an allergist, *n* (%)	
>10 years	33 (73.33%)
5–10 years	5 (11.11%)
<5 years	7 (15.56%)
Years of experience in managing patients with AIT, *n* (%)	
>10 years	29 (64.44%)
5–10 years	8 (17.78%)
<5 years	8 (17.78%)
Patients seen per month (n) mean (±SD)	298.11 (±151.36)
Proportion of polyallergic patients (%), mean (±SD)	59.89% (±19.98%)
Reason for consultation of polyallergic patients, %	
First visit	41.77%
Follow-up/control visit	58.23%
Geographical distribution, *n* (%)	
Cataluña and Islas Baleares	6 (13.33%)
Comunidad de Madrid, Castilla y León and Castilla-La Mancha	9 (20%)
Comunidad de Valencia and Castellón	5 (11.11%)
Bilbao, La Rioja, Navarra and Cantabria	7 (15.55%)
Málaga (Eastern Andalucía)	4 (8.89%)
A Coruña (Galicia)	4 (8.89%)
Sevilla and Huelva (Western Andalucía)	5 (11.11%)
Alicante and Murcia	5 (11.11%)

AIT, allergen immunotherapy; n, number of participants; SD, standard deviation.

a Number of experts who provided the requested descriptive information.

In general, the experts manage nearly 300 patients per month on average (range 60–900), with 60% being polyallergic, and most consultations were follow-up visits (58.23%) (Table 1).

### 3.2 Diagnosis of polyallergy

**Figure 1:**
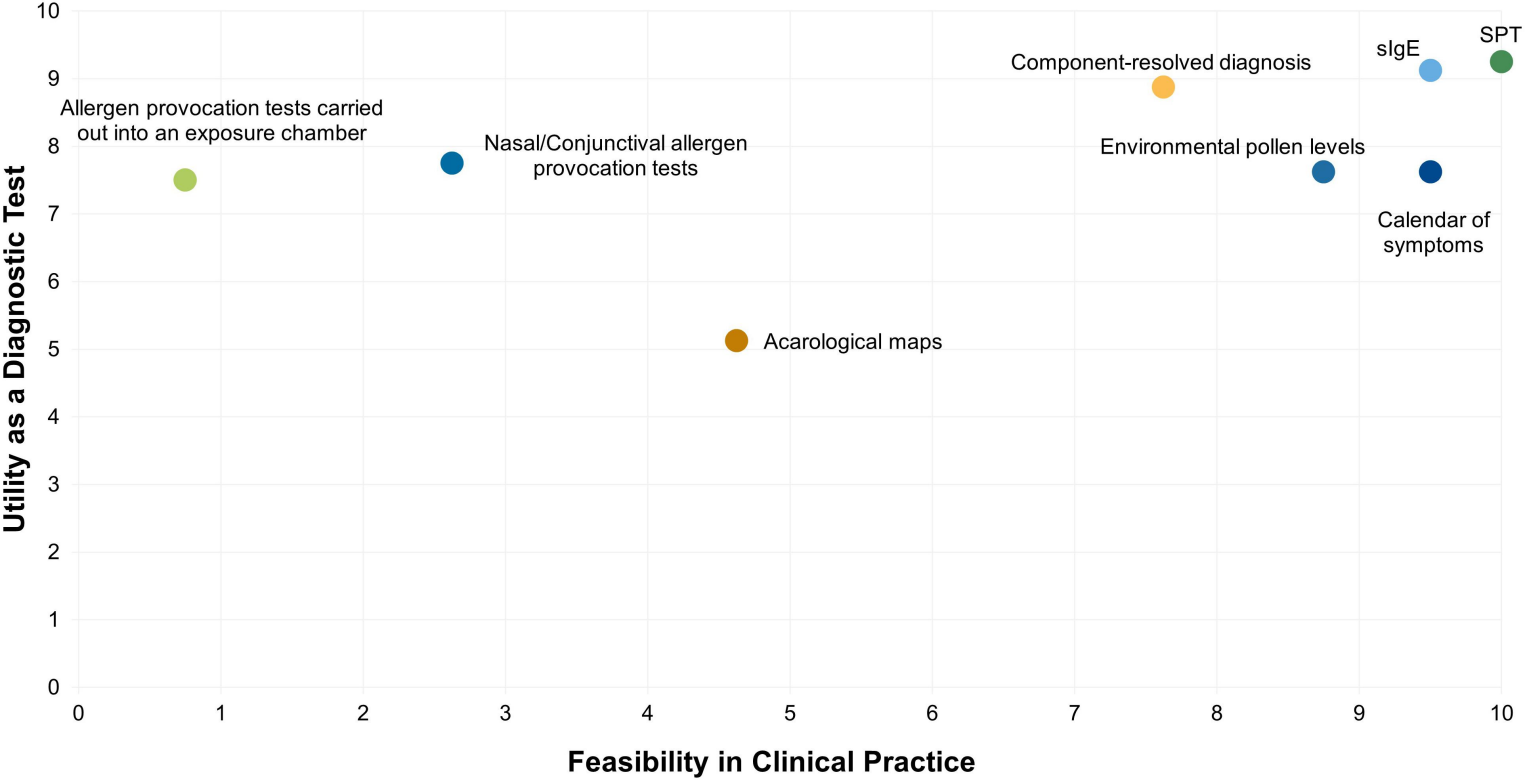
Scatter plot representing the utility and feasibility of each particular diagnostic test. *Y*-axis, 0 = not useful, and 10 = highly useful versus its feasibility in clinical practice; *X*-axis; 0 = nothing feasible and 10 = highly feasible for the diagnosis of polyallergy.

**Figure 2:**
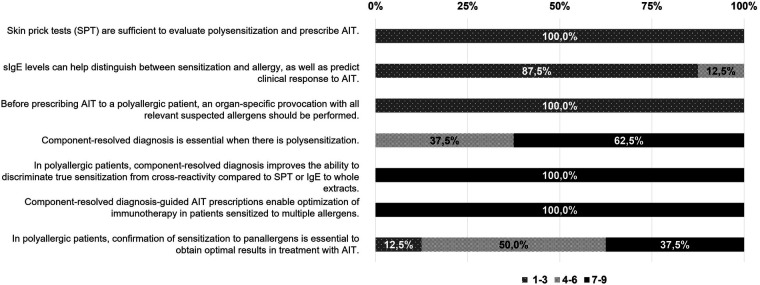
Degree of agreement or disagreement on the recommendations of the current evidence regarding the management of the polyallergic patient. The degree of agreement among participants has been evaluated according to a Liked scale from 1 to 9 (1–3, disagreement; 4–6 neutral; 7–9 agreement). The results are expressed as a percentage (%).

All experts agreed that it is essential for the allergist to establish a correct and thorough diagnostic pathway to prescribe the most appropriate therapeutic option. The allergy diagnosis presumes a pragmatic method, based on three essential steps: clinical record of symptoms history, skin prick test (SPT), and specific immunoglobulin E (sIgE) (Figures 1, 2). A detailed medical history will always be the key starting point to understand the patient's background, symptoms, and allergy triggers. Experts considered that an accurate diagnosis is affected by other factors, such as the allergist's experience and the patient's environment. SPT and sIgE are effective, accessible, and cost-efficient tools, that are usually the first step in diagnosis and guide the choice of subsequent complementary tests. In this context, experts agreed that component-resolved diagnosis is a particularly valuable tool due to its usefulness. However, it is not always feasible, as it is not routinely used or available in all allergy patient care centers (Figure 1). The majority (62.5%) of experts consider component-resolved diagnosis useful when there is polysensitization (Figure 2). All experts agreed that the use of component-resolved diagnosis is not strictly required in all cases, but it can enhance diagnostic precision and optimize AIT in polysensitized patients. On the other hand, the experts evaluated the usefulness of nasal and/or conjunctival provocation tests, as well as those carried out in exposure chambers, but their availability is low due to their demand of time and additional resources (Figures 1, 2). The calendar of symptoms was considered very useful in the diagnostic process by the experts in the REAL Project, but their effectiveness depends upon their correct completion by the patient (Figure 1).

In addition, pollen level registries were considered helpful, although their viability depends on the availability of a robust and reliable monitoring system. Similarly, acarological maps could potentially support diagnosis, but their utility is currently limited due to the lack of updated information (Figure 1).

### 3.3 Addressing the therapy approach with AIT

**Figure 3:**
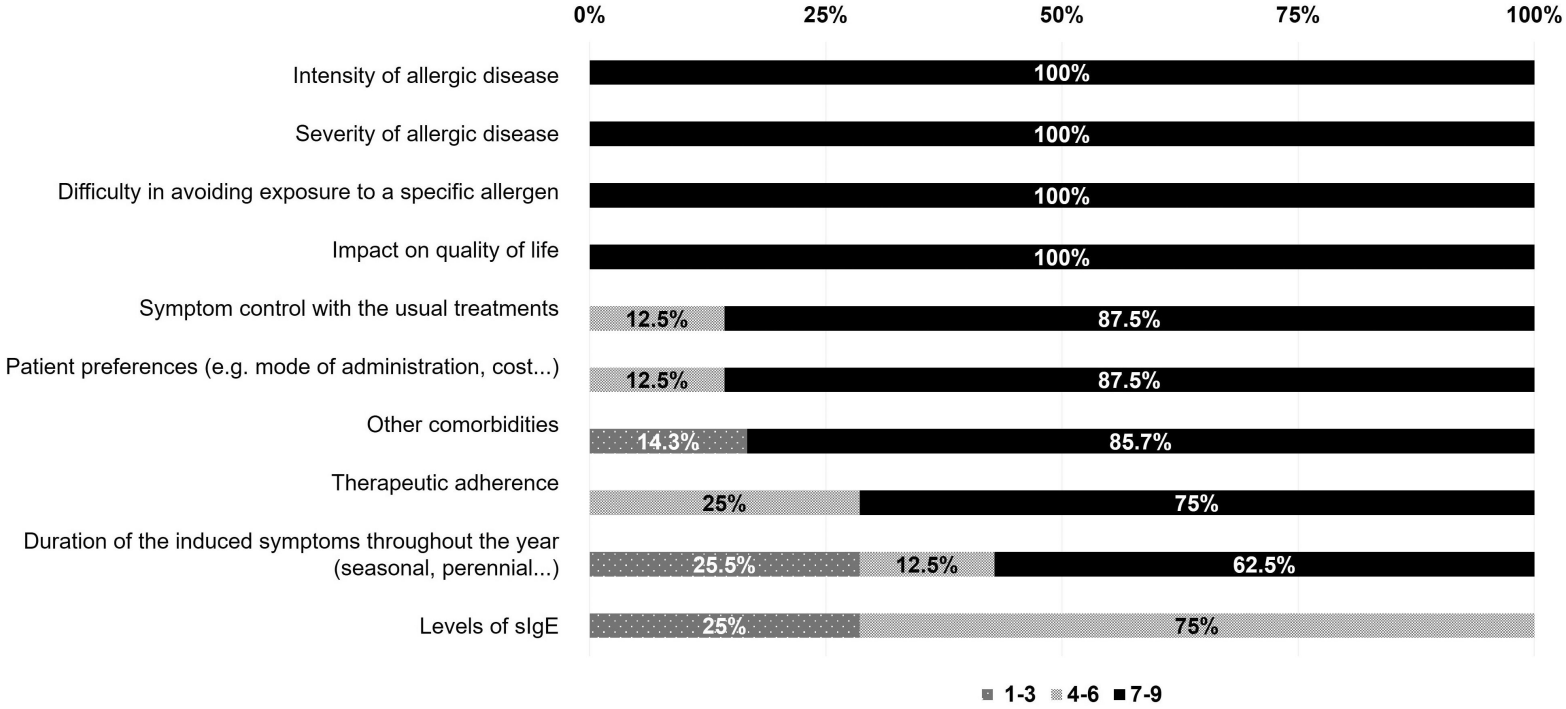
Relevance of each of the following items in selecting a therapeutic strategy when prescribing AIT based on mixtures from different allergenic sources in polyallergic patients. Agreement among participants was assessed using a 9-point Likert scale (1–3, disagreement/not relevant; 4–6 neutral; 7–9 agreement/relevant). Results are presented as percentages (%).

The REAL Project provides a qualified description of the pros and cons of selecting a particular therapy by keeping in focus the critical guiding factors chosen by the experts and based on the available evidence (Figure 3). When prescribing AIT in polyallergic patients, the presence of high symptom intensity or disease severity, as well as a clear impact on the patient's QoL were outlined as the most significant factors by 100% of experts, followed by patient preferences (87.5% of agreement), the adequate control of allergy with symptomatic treatments (87.5% of agreement), and the duration of such symptoms (62.5% of agreement). However, experts emphasized that neither allergy control nor symptom duration should limit the prescription of AIT, since the duration of symptoms throughout the year is not always correlated with disease severity, and symptom control does not preclude vaccination if symptoms are properly managed with medication (Figure 3). Avoiding exposure to a particular allergen is important, but not always achievable, so it was classified as less relevant.

Other factors that came up in the discussion were very relevant, including: the socio-economic conditions of each patient (habitat, profession, lifestyle), and the fact that age should not be considered a barrier for being treated with AIT. From the experts' perspective, AIT should therefore not be restricted to patients between the ages of 5 and 65 (data not shown).

### 3.4 The approach to polyallergy with AIT based on a mixture of different allergenic sources

The experts involved in the REAL Project discussed different topics related to the potential use of specific AIT prescriptions based on mixtures from different allergenic sources that are described below.

#### 3.4.1 Administration of AIT based on allergen mixtures from different allergenic sources

**Figure 4:**
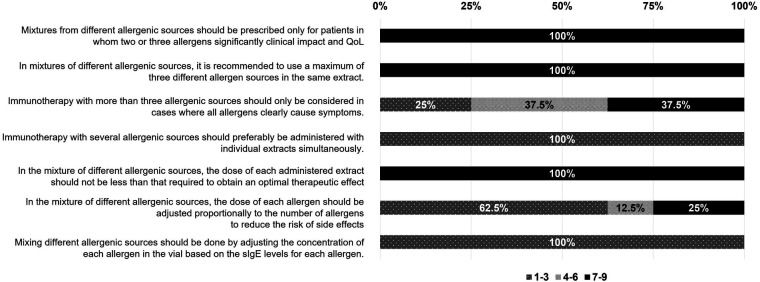
Administration of specific AIT based on mixtures from different allergenic sources in polyallergic patients. Participants expressed their level of agreement with the statements shown above. The degree of agreement among participants has been evaluated according to a Likert scale from 1 to 9 (1–3, disagreement; 4–6 neutral; 7–9 agreement). The results are expressed as a percentage (%).

The experts of the REAL Project generally recommended using a mixture of allergens in a single AIT treatment instead of using separated individual AIT extracts. Experts recommended the use of mixtures of up to three different allergenic sources with significant impact on symptoms and QoL, maintaining therapeutic concentrations of each allergenic source (Figure 4, Table 2). Although mixtures of polymerized allergen extract from two different allergenic sources are the most commonly used, the combination of three different allergenic sources is currently considered the recommended limit by the participating experts. In regions such as Balearic Islands, where there is a high proportion of polyallergic patients with symptoms caused by three allergenic sources, mixtures of three different extracts are more frequent.

**Table 2:** Selection of AIT based on mixtures of extracts from different allergenic sources and administration schedules in polyallergic patients.

Question	Response	Rational
Maximum number of allergens to be mixed	2 or 3 allergens from different allergenic sources to mix	The most common mixtures are generally based on 2 or 3 allergenic sources. The scientific evidence published to date (1) and clinical experience support the prescription of mixtures of up to 3 different allergenic sources, considering the clinical relevance of the allergenic sources in each patient.
		It is not excluded that, given the evolving needs of patients with polyallergy and the continuous progress in the development of new products, this recommendation may change in the future.
Type of extract	Polymerized extracts	Due to their stability and safety profiles, these are the type of extract recommended in AIT based on mixtures. In addition, this type of extract allows the mixing of different allergenic sources at high concentrations.
Administration route	Subcutaneous Immunotherapy (SCIT)	Subcutaneous administration is preferred mainly because of the extensive scientific evidence and clinical experience, in addition to other aspects such as availability, therapeutic adherence, and price.
Administration schedule	Perennial	Based on clinical experience, proven efficacy, and the higher frequency of perennial allergens, the perennial administration schedule is the most commonly used.
Extract concentration	Proportional mixing according to the number of extracts in the mixture	sIgE levels do not correlate with the patient's clinical condition.
		The mixture proportions should be adjusted according to the number of allergenic sources and not the levels of sIgE

However, 37.5% of the experts agreed to mix more than three allergens when these were considered undeniable causes of the patient's allergic symptoms, using polymerized extracts, provided that the mixtures were undiluted and expected to achieve the desired therapeutic effect, although such undiluted polymerized combinations are not currently available in clinical practice.

For 100% of the experts, the appropriate dose of each allergenic source in a mixture must be at its optimal concentration, which means that it must never be lower than that required to have the expected therapeutic effect. The concentration of each allergen should not be adjusted or diluted according to the levels of sIgE (Figure 4, Table 2).

#### 3.4.2 Criteria for the selection of AIT mixtures

Except for patient preferences, all experts believed that all the assessed criteria were relevant for choosing the different allergenic sources mixture (Table 3). A constant stability of the extract that can guarantee its activity over time, separately and in mixtures, as well as the use of polymerized extracts, proved to be essential (100% of agreement) for the final formulation of stable and safe mixtures.

**Table 3:** Relevant criteria for the selection of AIT mixtures from different allergenic sources.

Criterium	Score 1–3	Score 4–6	Score 7–9
Extract stability	0%	0%	100%
Polymerized extracts that allow the preparation of stable and safe mixtures	0%	0%	100%
Proven efficacy	0%	0%	100%
Safety	0%	0%	100%
Appropriate mixture/formulation (avoid mixtures with proteolytic activity)	0%	0%	100%
Quantification of major allergens	0%	25%	75%
Patient preferences	12.5%	50%	37.5%

The degree of agreement among participants has been evaluated according to a Likert scale from 1 to 9 (1–3, not relevant; 4–6 neutral; 7–9 relevant). The results are expressed as a percentage (%).

Likewise, the use of extracts with published scientific evidence on their efficacy and safety is highly recommended. Most experts (75%) valued the importance of quantifying at least a major allergen prior to modifying the extract (Table 3). Additional factors that should be taken into account according to the panel of experts were the number of allergenic sources (no more than three), the previous experience with these mixtures, the cost of the treatment, and the patient's geographical area (data not shown).

#### 3.4.3 Selection and administration routes and schedules for AIT based on mixtures of different allergenic sources

In AIT based on mixtures of different allergenic sources, the participating experts of the REAL Project were asked to select the most common recommended formulation for treating the polyallergic patient and the rationale for their decision The responses to this exercise are described in Table 2. For the purpose of this exercise, the mixture of *D. pteronyssinus/D. farinae* and the most common grasses was considered a two-component mixture. The current recommendation according to experts' opinion is to limit the mixture to a maximum of 3 allergenic sources in therapeutic doses, considering the clinical relevance of the allergens in each patient. This recommendation is based on the scientific evidence published to date (1) and their clinical experience. However, with the evolution of treatments and a better understanding of patient needs, the possibility of formulating mixtures with a greater number of allergens could be evaluated. The polymerized extracts were chosen by the experts for their stability and safety profile, which allows mixing different allergenic sources at high concentrations.

The preferred route of administration for the participating allergists in AIT was the subcutaneous route (Table 2). The panel explained that extensive scientific evidence and clinical experience, in addition to other aspects such as availability, therapeutic adherence, and treatment cost, were proposed to be the rationale for choosing this route of administration.

The most used administration schedule was the perennial (Table 2). The most important considerations for the perennial regimen were scientific evidence, the wide clinical experience available, and the relatively higher frequency of perennial allergens compared with seasonal allergens identified in routine clinical practice.

Experts concluded that the levels of sIgE were not useful to determine the concentration of the allergens in the mixture. This should then be done proportionally to the number of extracts to be included in the mixture.

**Figure 5:**
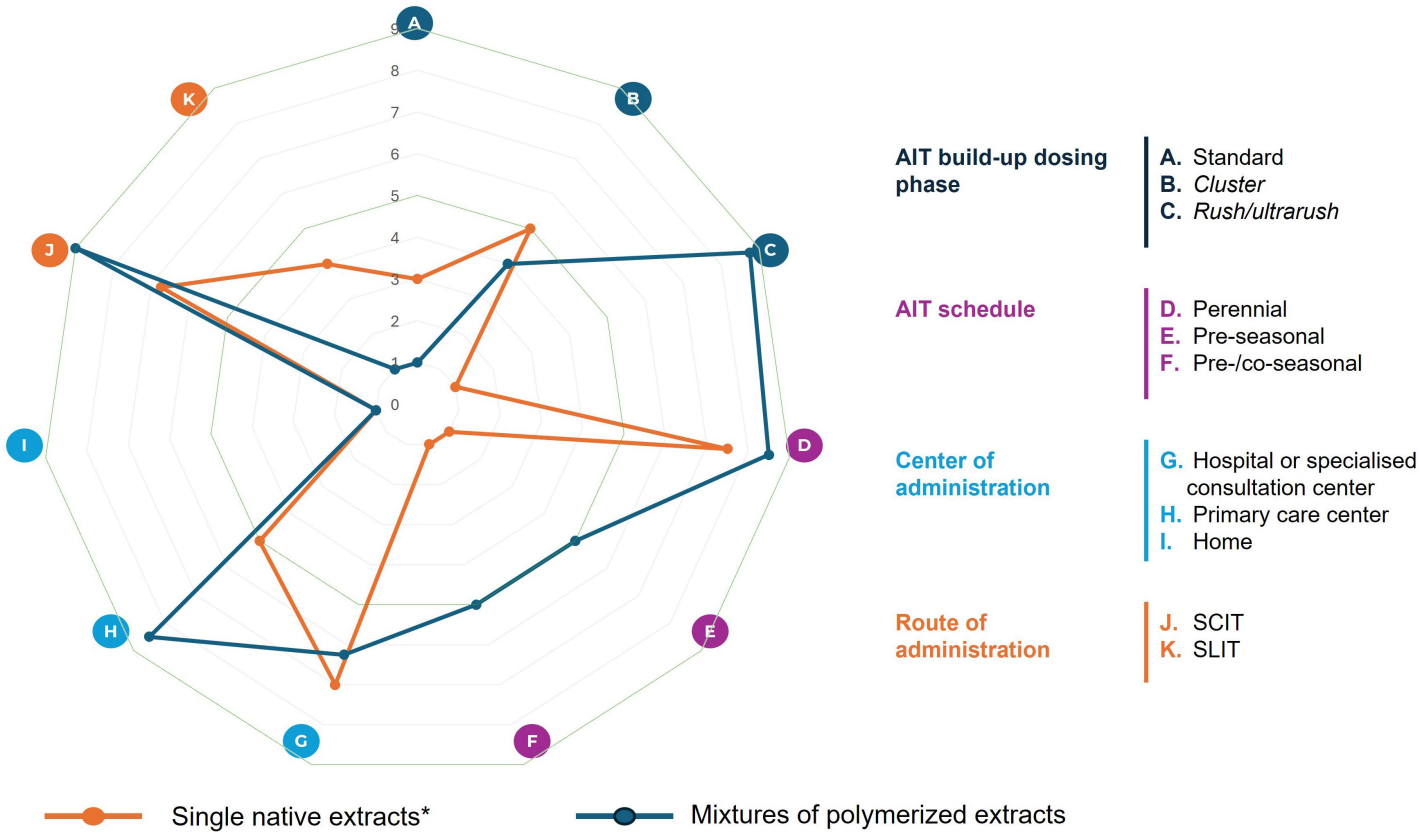
Spider maps of the scores (median values) given by participants to the different variables that can influence the administration patterns of specific allergen immunotherapy based on mixtures from different allergenic sources in polyallergic patients. AIT, allergen immunotherapy. *Despite the availability of native extract mixtures, experts do not recommend the use of AIT based on mixtures of different allergenic sources with native extracts. Therefore, the spider map was completed based on the recommended use of AIT with single native extracts and modified extracts.

Figure 5 represents a spider map, showing the median scores given by the experts in the discussion related to four topics (11 possible answers) about the administration routes and schedules of AIT based on different allergenic sources. A color code was used to distinguish preferences regarding the AIT formulation: single native extract or mixtures of polymerized allergens. Despite the availability of native extract mixtures, experts do not recommend using AIT with a mixture of native extracts. Thus, experts consider using polymerized extracts for AIT mixtures, as they offer greater stability, efficacy, and safety. The spider map was completed according to the recommended use of AIT with single native extracts and mixtures of polymerized allergens. The rush dosing schedule is the most frequently used for the initiation phase, allowing the maintenance dose to be reached more quickly without involving a greater number of adverse reactions. Additionally, perennial schedules are the most commonly used to ensure a continuous and effective treatment, as in some circumstances with specific allergens the pre/co-seasonal schedules can be also considered. Experts prefer the subcutaneous route over the sublingual one for administering the available mixtures, whether with native or polymerized extracts. In all meetings, a clear distinction was made between the place of administration for the initiation and maintenance phases of treatment. Initiation doses were generally administered in a hospital setting, while maintenance doses, regardless of whether they involved new vials, were typically administered at primary care centers in the case of subcutaneous immunotherapy (Figure 5).

#### 3.4.4 Criteria for assessing the response to treatment

**Figure 6:**
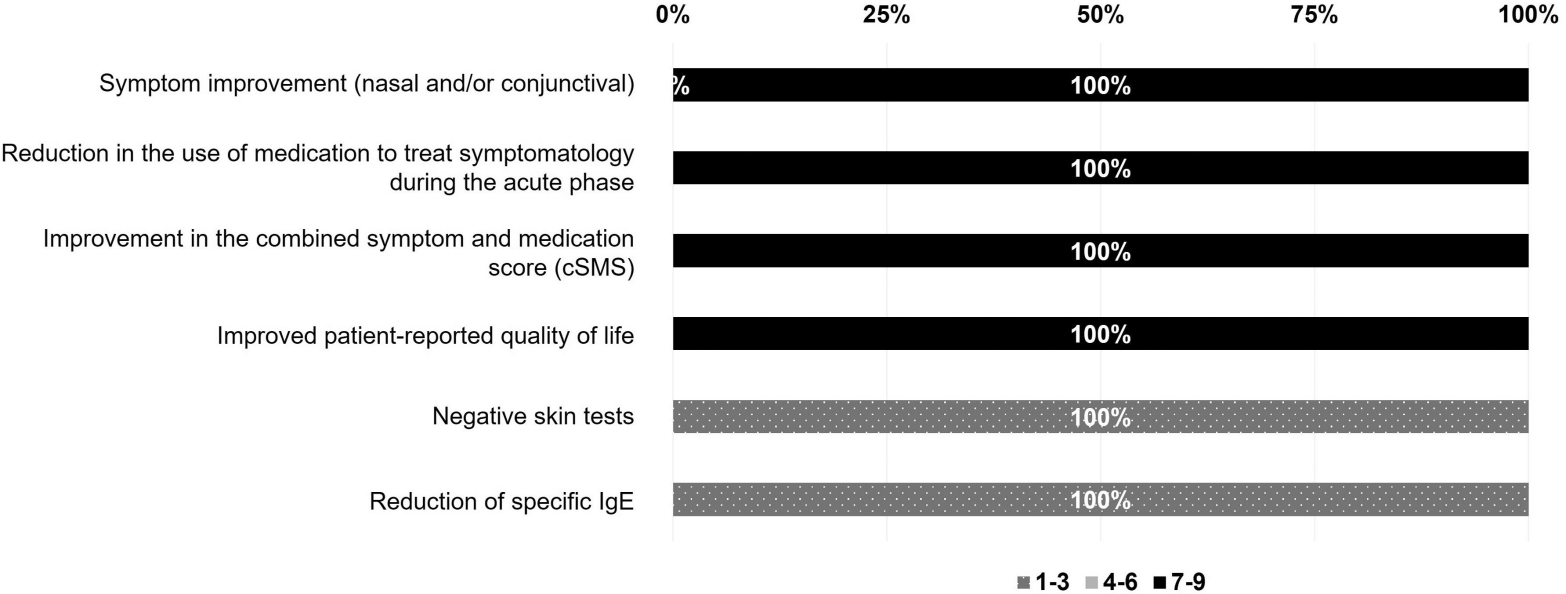
Degree of relevance of each of the following outcomes for the evaluation of the response to allergen immunotherapy. The degree of agreement among participants has been evaluated according to a Likert scale from 1 to 9 (1–3, not relevant; 4–6, neutral; 7–9, relevant). The results are expressed as a percentage (%).

The entire panel concurred (Figure 6) that the improvement of symptoms (nasal, conjunctival, and/or bronchial), the decrease of symptomatic medication used to control acute phase symptoms, and the improvement in the patient's self-reported QoL are the most important factors to consider when assessing the response to AIT. However, there is no need to repeat skin tests or sIgE, since this value does not always correlate with clinical symptoms.

Other factors described by the participating experts included: improvement in respiratory function, decrease in polyallergy-related work/school absenteeism, improvement in the overall comorbidities, improved therapeutic adherence, and the combined symptom and medication score (cSMS).

#### 3.4.5 Most frequently suggested formulations for mixtures based on different allergenic sources

Finally, experts of the REAL Project discussed the most commonly used mixtures or formulations in AIT in Spain. All formulations with mixtures from different allergenic sources were recommended if they were based on polymerized extracts, as they allow greater stability of the allergens, improving the safety and efficacy of the treatment. Furthermore, these mixtures are recommended in regions with multiple prevalent allergens, as they allow faster administration, improve adherence, and reduce treatment costs while maintaining safety. The choice of the most recommended mixtures depends on clinical experience and the specific need to mix certain allergens according to the different sensitization profiles of polyallergic patients. However, limited experience with some combinations in certain geographical areas may influence their use among experts.

## 4 Discussion

Although AIT with polymerized allergen extract mixtures of different allergenic sources is commonly used in clinical practice in Spain, there is currently a lack of consensus or published guidelines on how to manage polyallergic patients using this approach. In this context, allergists seek a widely approved and conclusive management strategy for the optimization of AIT prescription.

This is particularly relevant in Spain, where geographic and environmental diversity leads to complex sensitization profiles. As described by Martínez-Cañavate et al., different regions of Spain present heterogeneous patterns of aeroallergen sensitization, influenced by environmental factors, climate conditions, and urbanization levels (26). Furthermore, recent molecular mapping studies (27), strong found regional differences across Europe regarding IgE sensitizations to respiratory allergens which can be attributed to the climate in certain areas. These region-specific patterns complicate the interpretation of sensitization profiles and add an additional layer of complexity to clinical decision-making, making the management of polyallergic patients especially challenging.

In response to this need, the REAL Project was designed as a pioneering initiative to establish practical, evidence-based recommendations for the use of AIT with mixtures from different allergenic sources in Spain, based on the opinion of expert allergists. It should be noted that the selection criteria of the participating allergists ensure that panelists have demonstrated expertise and knowledge to assess the scope of the project, with most of them having more than 10 years of experience in managing AIT for polyallergic patients and an average of 180 polyallergic patients per month. The diverse distribution across different types of healthcare settings and regions across Spain further reinforces the applicability of the results.

The Workmat® methodology employed in the present study has encouraged debate among experts, who were able to share their professional experience and the differential approach strategies to polyallergy across the 8 regions participating in the project. Following these discussions, the degree of agreement and relevance observed in the results of this study has revealed that we are facing three significant challenges: (i) polyallergy has increased progressively making the use of AIT with mixtures based on different allergenic sources an increasingly necessary and effective option; (ii) the known benefits of using mixtures vs. individual extracts; and (iii) the efficacy and safety of this type of mixtures.

### 4.1 Map of allergen sensitization and diagnosis of polyallergic patients

Polysensitization is an immunological event that is relevant from an epidemiological and clinical point of view (31) and is typically more common than monosensitization in general population surveys conducted worldwide using standard panels of allergens (32–35). Unsurprisingly, polysensitization accounts for 60%–80% of patients who consult an allergist (34, 36, 37). In 2022, a study conducted in Spain obtained a map of sensitization to allergenic sources based on clinical history, allergic symptoms, and SPT results. This study demonstrated that 79% of allergic patients from different regions of Spain showed sensitization to two or more allergenic sources (9).

Participants of the REAL Project highlighted that among patients with allergic symptoms, an increasing number of subjects are polysensitised and most importantly polyallergic. The severity of symptoms and the prevalence of asthma are also rising (31, 38). The burden of allergic symptoms is therefore increasing, with a negative impact on patients' overall health (39). Because of this growing trend of polyallergy, panelists emphasize the relevance of meeting patients' needs, which involves having an accurate identification of their sensitization profile and a proper diagnosis.

Importantly, Spain presents a unique and particularly complex allergenic landscape. Unlike other countries where a small number of allergenic sources may predominate, Spanish patients are frequently exposed to a wide range of allergens due to geographic, climatic, and environmental diversity (26, 27). This results in regional differences in the prevalence and intensity of sensitization patterns. This complexity of the allergenic exposome scenario makes the diagnostic process especially challenging and highlights the need for tailored strategies adapted to local sensitization profiles.

Given this context, the panelists emphasized the importance of accurately identifying each patient's sensitization profile and establishing a proper diagnosis. When sensitization to two or more allergens is present, it is important to identify the allergenic source(s) with the greatest impact on QoL. The allergists' main responsibility is to determine which sensitizing allergens are relevant concerning the clinical symptoms of the allergy. A detailed clinical history alone is still often sufficient for an etiological diagnosis, being frequently enough to identify the clinically relevant allergen or allergens responsible for the allergic respiratory disease. As an example, it has been reported that the clinical history alone had a predictive value of 82%–85% for seasonal allergens and at least 77% for perennial allergens, but when both sIgE and SPT are available these values may increase to 97%–99% (40).

In the present study, participant allergists agree that an adequate diagnosis requires correlating clinical findings with SPT and/or sIgE quantification, with component-resolved diagnosis recommended to distinguish genuine sensitizations from cross-reactivity in polysensitized patients. According to what has been published, panelists outline the limited value of SPT in recognizing some relevant allergens (41). However, SPT is commonly used in daily clinical practice because of its simplicity, sensitivity and positive predictive value in quickly identifying IgE sensitization to inhalant allergens (42).

The first step to properly approach polysensitization involves differentiating between genuine or primary sensitization and cross-sensitization. Component-resolved diagnosis may help allergists to identify clinically relevant allergens and distinguish genuine polysensitization (“co-sensitization”) from polysensitization due to cross-reactivity (“cross-sensitization”). Accurate identification of the allergens responsible for sensitization may help allergists develop personalized etiological AIT treatments (43, 44).

Following the identification of the most clinically relevant allergens based on clinical history and the complementary test of choice (SPT, sIgE, component-resolved diagnosis), the next decision will be how to treat a polyallergic patient. AIT is a recognized effective therapy for respiratory allergy with a unique potential ability to modify the natural course of the disease and induce long-term clinical benefits, that can persist for years after discontinuation of treatment (45). It is necessary then to know which factors may influence the choice of treatment. The allergist evaluates the clinical history based on the presence, severity, and duration of seasonal and/or perennial symptoms and their impact on Quality of Life (QoL). The causal relationship between the most troublesome clinical symptoms and the relevant allergens appears to be a key factor to the experts. Consistent with this, a previous Spanish Delphi consensus on the diagnosis and treatment of polyallergy—with the participation of 62 allergists— stated that AIT prescriptions should be based on scientific evidence and only given if relevant allergens have been identified that correspond to symptom severity (1).

### 4.2 The benefits of using mixtures vs. individual extracts in polyallergy

Based on previously published experiences (16, 17, 46), this project shows that allergen mixtures based on different allergenic sources are a valid alternative when two or more allergens that may or may not overlap in time, have a comparable and substantial impact on the patient´s symptoms and QoL. Furthermore, these allergen mixtures, especially those at undiluted concentrations, offer several advantages over AIT with simultaneous individual extracts, as they allow the treatment of multiple clinically relevant sensitizations in a single application, simplifying the treatment without compromising its efficacy and saving both time and costs.

Allergists in the US believe that addressing as many sensitizations or allergies in a patient as possible offers significant clinical benefits (46). More recently, a survey was conducted online with allergy-practicing clinicians in 19 countries worldwide in 2016. A total of 1,029 eligible clinicians prescribing AIT provided information on their approach to AIT, including managing polyallergic patients; 98% of the responders reported the treatment of polyallergic patients. In this regard, country-specific differences were detected: 58% of those polyallergic patients received AIT treatment for a single allergen, while 42% were prescribed AIT with multiple allergens, including either mixture (24%) or single allergen AIT (18%) (22). These findings underscore the importance of maintaining a patient-centered approach in AIT preparations, in alignment with regulatory authority recommendations. It is essential that AIT products meet high-quality standards and are supported by evidence of efficacy and safety. Based on our discussions, 100% of the participants agreed to use up to three distinct allergens in a single extract when dealing with mixtures of different allergenic sources; and, if possible, choose to use mixtures in a single vaccine over several individual extracts.

In this regard, prescriptions of AIT with three or more different allergenic sources are rare in European countries other than Spain (47). In observational surveys in France, only 1.1% of AIT prescriptions contained three or more allergenic sources (48). The recommendation when using mixed AIT formulations is maintained at a maximum of three different allergenic sources, but it may change in the future due to the increase in the prevalence of polyallergy and the development of new treatments.

Nonetheless, when treating polyallergy there is a trend in some European countries towards prescribing AIT with a single allergen or with several allergens, but separately. The appropriate dose of each allergen in a mixture based on different allergenic sources should be at a therapeutic concentration to prevent the dilution of each allergen extract below the therapeutic dose. This approach aligns with the principles of the EMA, which emphasizes the use of the minimum number of allergens in mixtures with justified proportions of individual allergens (49). This restriction might be explained based on several premises. In addition to the aforementioned dilutional effect, there is a possibility of degradation due to the enzymatic activity of some extracts, and the difficulty in demonstrating the efficacy of many allergens (49, 50). Additionally, it was advised to minimize the number of allergens in the mixture according to the patient's polyallergic profile and the use of extracts with proven scientific evidence of their efficacy and safety.

In this context, the use of polymerized allergen extracts in the treatment of polyallergic patients is emerging as a promising therapeutic strategy, overcoming the limitations traditionally stated by the EMA with respect to the mixture of allergens. Polymerization of extracts significantly reduces the binding capacity of specific IgE to allergens (antigenicity) without altering the ability to induce an adequate immune response (immunogenicity) (4, 51, 52). This allows the concentration of allergens to be increased without increasing the risk of adverse reactions (1, 51). In addition, this process inactivates the enzymatic activity of the extracts, which makes it possible to combine allergens from different sources, even those with high enzymatic activity that, under normal conditions, would be incompatible. In summary, polymerized extracts allow the simultaneous administration of multiple allergens at optimal concentrations within a single formulation, eliminating the need for dilution (49, 53).

Published investigations of real-world data coming from almost 3,000 children and adolescents with rhinoconjunctivitis with/or without asthma suggest that AIT with mixtures of depigmented and polymerized extracts is efficacious and well tolerated (50).

This is particularly relevant in polyallergic patients, where the use of stable and standardized mixtures is crucial to maintain the effectiveness of the treatment over time and to avoid loss of potency due to extract incompatibility. Therefore, to provide optimal treatment, the panel of allergists recommended using polymerized allergen extracts—such as depigmented polymerized extracts—that have a very reduced proteolytic activity, avoiding allergen mixtures that may interact (53).

The route of administration of AIT should be customized for each patient using a patient-oriented approach (16). When deciding between the sublingual immunotherapy (SLIT) or the subcutaneous immunotherapy (SCIT) routes, there are many factors to consider, such as availability, treatment cost, adherence, clinical experience, and scientific evidence (54). All these factors have made SCIT the preferred route for our experts. Overall, numerous studies provide evidence supporting the effectiveness of SCIT in the management of allergic rhinitis with or without asthma. These studies have primarily focused on the relief of allergic symptoms and the enhancement of QoL, the decrease in exacerbations and improvement in lung function; and reducing the use of medications for symptomatic treatment (54–57). Similarly, numerous studies have demonstrated the short and long-term efficacy of SLIT in allergic rhinitis with or without asthma in both adult and pediatric patients (54, 58, 59), but this was not the best-considered route of administration in terms of adherence, where the experts were clearly in favor of SCIT.

The administration of AIT, including that with different allergenic sources, involves a two-phase scheme: an initiation phase using one of three protocols (i.e., standard, *cluster*, or *rush)*; and a maintenance phase following a perennial, pre-seasonal, or pre/co-seasonal schedule. This study supports the use of polymerized extracts initially administered in a *rush* build-up dosing phase, which allows the maintenance dose to be reached more quickly without involving a greater number of adverse reactions (60), followed by a perennial maintenance schedule, which is the most commonly used to ensure continuous and effective treatment.

### 4.3 Efficacy and safety of immunotherapy based on mixtures of different allergenic sources

The use of polymerized allergen extracts has improved both the efficacy and safety of AIT in polyallergic patients; therefore, it is essential to conduct more individualized evaluations of the response to treatments, considering the type of extract and the mixtures.

The most relevant features to assess the response to AIT are the improvement of either nasal, conjunctival, and/or bronchial symptoms, the reduction in the amount of medication, and the enhancement of the patient's self-reported QoL. Prior studies indicate that physicians can prescribe mixed multi-allergen immunotherapy with confidence in polysensitized and polyallergic patients by focusing on clinical/QoL relevance and safety (5). Pfaar et al. conducted a 2-year multicenter, double-blind, placebo-controlled trial of SCIT with mixed depigmented and polymerized birch and grass pollen extract in 285 patients with allergic rhinoconjunctivitis who experienced symptoms during both birch and grass pollen seasons (61). Compared to placebo, SCIT resulted in a significant 19.4% reduction in the cSMS, with a good safety profile. An observational, retrospective, multicentric study of 40 pediatric patients with rhinitis/rhinoconjunctivitis, with or without controlled asthma, showed that an undiluted mixture of depigmented and polymerized grass/olive (Olea europaea) allergens was effective in both perennial and pre/co-seasonal schedules, and therefore suitable for polyallergic patients (17). In this report, the response of AIT revealed a significant improvement in symptoms, QoL, and global perception of the disease, whereas safety profiles were good and similar in both schedules.

It is known that as the number of patient sensitizations increases, the severity of associated respiratory diseases escalates (37). Single multiallergen AIT must demonstrate effectiveness while upholding a solid safety profile; in contrast, multiple monoallergen immunotherapy can be associated with poor treatment adherence and high costs. Another retrospective observational study investigated the effect of using a 2-pollen single undiluted multiallergen SCIT (duration, 1.8 years) in 97 patients (median age 32 years) in routine clinical practice in Spain (62). SCIT treatment, including combinations of Grass-mix with *Olea europaea*, *Parietaria judaicaspp*, *Cupressus arizonica*, *Platanus acerifolia*, or *Salsola kali* or *Olea europaea* with *Parietaria judaica*, *Cupressus arizonica* or *Salsola kali*, was effective and safe in both children and adults. This was a well-tolerated dosing scheme, with little systemic reactions (6 out of 97 cases), none of which led to treatment discontinuation, showing that it is a suitable option for the treatment of polyallergic patients.

AIT has therefore proven to be safe, although some factors are dependent on the patients and/or the extracts. Furthermore, the administration site may affect tolerability (57, 63). SLIT has shown less incidence of systemic reactions than SCIT: the incidence of SLIT systemic reactions is 0.0045% (64). The most common reactions are local and usually appear in the first days of treatment. The rate of systemic reactions in SCIT is between 0.1% and 1.56% per administration, and they usually occur within the first 30 min after application. The frequency of anaphylactic reactions in *rush* or *ultrarush* schedules without prior medication, as well as delays in the administration of SLIT, are risk factors for systemic reactions during the initiation phase (57).

Notably, the participating allergists consider that all formulations appear to be recommendable if they are stable, safe, and effective, including allergens that impact the QoL and symptoms, and do not have proteolytic activity. Only the lack of experience relative to a particular AIT mixture in a certain geographic region may condition to recommend its use to treat polyallergy. The provided combinations were deemed advisable for various reasons: (i) these common allergens are usually responsible for the symptoms observed in each particular geographical area; (ii) the use of these allergen mixtures in AIT makes it possible to provide a less expensive therapy, facilitating the prescribing process and enhancing treatment adherence; and (iii) these mixtures were highly believed to be safe for the patient.

In this context, it is important to note that certain allergenic sources, although not highly prevalent in other European countries, are frequently involved in the development of allergic symptoms in Spain—either nationally (e.g., *Olea europaea*, *Parietaria judaica*) or in specific regions (e.g., *Salsola kali*, *Cupressus arizonica*) (65, 66). These complex and region-specific sensitizations patterns are often managed through mixed allergen formulations, as long as they are stable and meet safety standards. Therefore, stability data on allergen mixtures is particularly relevant when considering the use of individualized combinations adapted to the sensitization profiles observed in different Spanish regions.

Although the absence of provocation data precludes establishing causal relationships or definitive therapeutic hierarchies, the collective experience of the experts provides a pragmatic and contextually valid framework for guiding clinical decision-making in the Spanish setting.

In this study, we gathered expert input on the use of such mixtures in polyallergic patients, providing practical guidance for their application. The recommendations derived from this project are based on expert judgement and real-world clinical practice rather than on data from provocation tests or other controlled experimental assessments.

While qualitative, non-randomized methods have inherent limitations, including potential selection bias and unequal regional representation, the consistency of expert opinions across Spain supports the validity of the findings. These recommendations reflect the Spanish clinical context, shaped by geographic and environmental diversity, regulatory frameworks, and the widespread use of polymerized extracts. Although direct extrapolation to other countries may be limited, the structured Workmat® methodology and the expert reasoning behind these recommendations may offer relevant insights for regions that face similarly complex polysensitization patterns, supporting the integration of evidence with routine clinical practice.

## 5 Conclusions

In summary, our findings highlight the need to consider AIT based on mixtures of polymerized extracts from different allergenic sources, whenever multiple sensitizations have been demonstrated to be clinically relevant in polyallergic patients. This therapeutic strategy becomes particularly important when polysensitization has a significant impact on the patient's QoL, allowing a more comprehensive and personalized approach to treatment.

The selection of these mixtures should be based on criteria of stability, efficacy, and safety with a crucial emphasis on the use of polymerized extracts. Mixtures of allergens from different allergenic sources should be limited to a maximum of three with a relevant clinical impact. The scheme of choice for AIT recommended by the experts is a rush build-up phase and the perennial schedule; it is essential to ensure that each extract is included in the therapeutic dose, considering the total number of extracts in the mixture, to achieve an effective therapeutic response. The degree of response to the treatment should be evaluated according to the improvement of symptoms, the reduction in the use of medication, and the positive impact on QoL.

Although mixtures with polymerized extracts are generally recommended, the lack of experience with certain combinations in different regions may influence their use.

Ultimately, the REAL Project recommendations not only support a more effective management of polyallergic patients but also highlight the urgent need to adapt European guidelines to reflect the practical challenges and allergenic diversity encountered in real-world clinical settings, particularly in countries with complex and diverse polysensitization patterns like Spain.

## Data Availability

The raw data supporting the conclusions of this article will be made available by the authors, without undue reservation.
