# Supplementary material for: The Spanish REAL project: expert allergist guidance on allergen immunotherapy based on mixtures from different allergenic sources
Source: Front Allergy. 2026 Jan 30;7:1736462. doi: 10.3389/falgy.2026.1736462 (PMC12900685; doi:10.3389/falgy.2026.1736462)
Supplement: Supplementary file 1 [file Table1.docx]

**Supplementary Table 1. Regional Workmat® agreement scores (1–9 scale) for each statement.**

| **Exercise** | **Region 1** | **Region 2** | **Region 3** | **Region 4** | **Region 5** | **Region 6** | **Region 7** | **Region 8** |  |  |  |  |  |  |  |  |  |  |
| --- | --- | --- | --- | --- | --- | --- | --- | --- | --- | --- | --- | --- | --- | --- | --- | --- | --- | --- |
| **Utility/feasibility of diagnostic tests and/or tools**  *Place the following tools on the grid according to their usefulness in the diagnosis of the polyallergic patient and their feasibility in clinical practice. (Indicate each letter on the grid based on the score assigned for usefulness—where 0 = not useful at all and 10 = very useful—and feasibility—where 0 = not feasible at all and 10 = very feasible.)* | | | | | | | | |  |  |  |  |  |  |  |  |  |  |
| *Utility:* | | | | | | | | |  |  |  |  |  |  |  |  |  |  |
| Specific immunoglobulin E (sIgE) (in vitro) | 9 | 9,75 | 8,25 | 9,25 | 9,75 | 8,5 | 9,25 | 6,75 |  |  |  |  |  |  |  |  |  |  |
| Component-resolved diagnosis | 9 | 9,5 | 6,5 | 8,75 | 9,75 | 9 | 7,5 | 8,75 |  |  |  |  |  |  |  |  |  |  |
| Skin prick tests (SPT) (in vivo) | 9 | 10 | 9,75 | 8,5 | 9,75 | 8 | 9,5 | 8,5 |  |  |  |  |  |  |  |  |  |  |
| Nasal and/or conjunctival allergen provocation tests | 7,5 | 8,25 | 8 | 8,75 | 9,75 | 7,5 | 3,5 | 3,75 |  |  |  |  |  |  |  |  |  |  |
| Allergen provocation tests carried out into an exposure chamber | 8,5 | 8 | 2,25 | 9 | 9,75 | 7 | 7 | 4,75 |  |  |  |  |  |  |  |  |  |  |
| Calendar of symptoms | 8,5 | 7,5 | 7,25 | 7,5 | 7,75 | 8 | 7,5 | 8 |  |  |  |  |  |  |  |  |  |  |
| Environmental pollen levels | 8 | 6 | 7,25 | 7,5 | 9,25 | 7,5 | 8,5 | 7,75 |  |  |  |  |  |  |  |  |  |  |
| Acarological maps | 5 | 3,5 | 6,5 | 1,25 | 5,25 | 6,5 | 7,25 | 2,75 |  |  |  |  |  |  |  |  |  |  |
| *Feasibility:* | | | | | | | | |  | 9,75 | 8,25 | 9,25 | | 9,75 | 8,5 | 9,25 | 6,75 |  |
| Specific immunoglobulin E (sIgE) (in vitro) | 9,5 | 9 | 10 | 9,5 | 9,25 | 9,5 | 9,75 | 9,25 |  |  |  |  |  |  |  |  |  |  |
| Component-resolved diagnosis | 6,75 | 6,75 | 5 | 9 | 8 | 8 | 7,25 | 9,25 |  |  |  |  |  |  |  |  |  |  |
| Skin prick tests (SPT) (in vivo) | 10 | 10 | 9,75 | 10 | 10 | 9,5 | 10 | 10 |  |  |  |  |  |  |  |  |  |  |
| Nasal and/or conjunctival allergen provocation tests | 3,25 | 3,5 | 3 | 2 | 1,75 | 5,5 | 2 | 2,25 |  |  |  |  |  |  |  |  |  |  |
| Allergen provocation tests carried out into an exposure chamber | 1 | 0,5 | 0,25 | 0 | 5 | 1,5 | 2 | 0,25 |  |  |  |  |  |  |  |  |  |  |
| Calendar of symptoms | 4,875 | 10 | 7,25 | 9,5 | 9,75 | 9,5 | 10 | 7 |  |  |  |  |  |  |  |  |  |  |
| Environmental pollen levels | 8 | 10 | 9 | 9,5 | 9 | 8,5 | 7 | 6,25 |  |  |  |  |  |  |  |  |  |  |
| Acarological maps | 6,5 | 3,5 | 1 | 5 | 8,5 | 5 | 1,5 | 4,25 |  |  |  |  |  |  |  |  |  |  |
| **Factors influencing the choice of the therapeutic strategy.**  *When prescribing allergen immunotherapy (AIT) based on mixtures from different allergenic sources in polyallergic patients, please indicate the degree of importance that each of the following factors should have in the choice of the therapeutic strategy. (Rate the importance from 1 to 9, where 1 = “not important at all” and 9 = “very important”).* | | | | | | | | |  |  |  |  |  |  |  |  |  |  |
| Intensity of allergic disease | 8,5 | 9 | 8,5 | 8,5 | 9 | 9 | 9 | 9 |  |  |  |  |  |  |  |  |  |  |
| Severity of allergic disease | 8,5 | 8,5 | 8,5 | 8,5 | 9 | 9 | 8 | 9 |  |  |  |  |  |  |  |  |  |  |
| Difficulty in avoiding exposure to a specific allergen | 8,5 | 7,5 | 7,5 | 8,5 | 8,5 | 9 | 8 | 8 |  |  |  |  |  |  |  |  |  |  |
| Impact on quality of life | 8,5 | 8 | 9 | 8 | 9 | 9 | 9 | 8,5 |  |  |  |  |  |  |  |  |  |  |
| Symptom control with the usual treatments | 8 | 7,5 | 5 | 8 | 8 | 9 | 7 | 7 |  |  |  |  |  |  |  |  |  |  |
| Patient preferences (e.g. mode of administration, cost...) | 8,5 | 7 | 8,5 | 9 | 9 | 8 | 6,5 | 8,5 |  |  |  |  |  |  |  |  |  |  |
| Other comorbidities | 8 | 8 | 2 | 9 | 8,5 | 9 |  | 7,5 |  |  |  |  |  |  |  |  |  |  |
| Therapeutic adherence | 7,5 | 9 | 9 | 4 | 8 | 5,5 | 8 | 8,5 |  |  |  |  |  |  |  |  |  |  |
| Duration of the induced symptoms throughout the year (seasonal, perennial...) | 8,5 | 8,5 | 3 | 8,5 | 1 | 8 | 6,5 | 8,5 |  |  |  |  |  |  |  |  |  |  |
| Levels of sIgE | 6 | 5 | 1,5 | 5 | 6 | 4,5 | 4 | 3 |  |  |  |  |  |  |  |  |  |  |
| **Treatment of polyallergy with AIT based on mixtures from different allergenic sources**  *In the treatment of polyallergy with AIT based on mixtures from different allergenic sources, please indicate your level of agreement with the following statements. (Rate your agreement from 1 to 9, where 1 = “strongly disagree” and 9 = “strongly agree”).* | | | | | | | | |  |  |  |  |  |  |  |  |  |  |
| Mixtures from different allergenic sources should be prescribed only for patients in whom two or three allergens significantly clinical impact and QoL. | 7 | 9 | 9 | 9 | 9 | 9 | 8 | 8,5 |  |  |  |  |  |  |  |  |  |  |
| In mixtures of different allergenic sources, it is recommended to use a maximum of three different allergen sources in the same extract. | 8 | 8,5 | 9 | 8,5 | 9 | 8,5 | 9 | 8,5 |  |  |  |  |  |  |  |  |  |  |
| Immunotherapy with more than three allergenic sources should only be considered in cases where all allergens clearly cause symptoms. | 7,5 | 3,5 | 1,5 | 9 | 6 | 5 | 4 | 9 |  |  |  |  |  |  |  |  |  |  |
| Immunotherapy with several allergenic sources should preferably be administered with individual extracts simultaneously. | 3,5 | 1 | 1 | 3 | 1 | 1,5 | 1,5 | 2 |  |  |  |  |  |  |  |  |  |  |
| In the mixture of different allergenic sources, the dose of each administered extract should  not be less than that required to obtain an optimal therapeutic effect. | 8,5 | 9 | 9 | 9 | 9 | 9 | 9 | 9 |  |  |  |  |  |  |  |  |  |  |
| In the mixture of different allergenic sources, the dose of each allergen should be adjusted  proportionally to the number of allergens to reduce the risk of side effects. | 5 | 1,5 | 1 | 1 | 9 | 1,5 | 1,5 | 8,5 |  |  |  |  |  |  |  |  |  |  |
| Mixing different allergenic sources should be done by adjusting the concentration of each  allergen in the vial based on the sIgE levels for each allergen. | 1,5 | 1 | 1,5 | 1 | 1 | 2 | 1,5 | 1 |  |  |  |  |  |  |  |  |  |  |
| **AIT administration**  *In the administration of AIT based on mixtures from different allergenic sources, indicate how the following treatments should be administered, using the markers of the indicated colors. (Rate your preference for each regimen from 1 to 9, where 1 = “strongly disagree” and 9 = “strongly agree”).* | | | | | | | | |  |  |  |  |  |  |  |  |  |  |
| Single native extracts | | | | | | | | |  |  |  |  |  |  |  |  |  |  |
| *AIT build-up dose phase:* | | | | | | | | |  |  |  |  |  |  |  |  |  |  |
| Standard | 5 | 5 | 1 | 6,25 | 1 | 1 | 5 | 1 |  |  |  |  |  |  |  |  |  |  |
| Cluster | 5 | 4 | 5 | 7,75 | 9 | 1 | 3,5 | 9 |  |  |  |  |  |  |  |  |  |  |
| Rush/ultrarush | 1 | 5 | 1,5 | 1 | 1 | 1 | 1 | 1 |  |  |  |  |  |  |  |  |  |  |
| *AIT schedule* | | | | | | | | |  |  |  |  |  |  |  |  |  |  |
| Perennial | 7 | 9 | 5 | 9 | 9 | 1 | 5 | 8 |  |  |  |  |  |  |  |  |  |  |
| Pre-seasonal | 5 | 2 | 1 | 1 | 1 | 1 | 1 | 1,5 |  |  |  |  |  |  |  |  |  |  |
| Pre-/co-seasonal | 5 | 1,5 | 1 | 5 | 1 | 1 | 1 | 1 |  |  |  |  |  |  |  |  |  |  |
| *Center of administration:* | | | | | | | | |  |  |  |  |  |  |  |  |  |  |
| Hospital or specialised consultation center | 1 | 9 | 5 | 9 | 9 | 1 | 5 | 9 |  |  |  |  |  |  |  |  |  |  |
| Primary care center | 9 | 7 | 5 | 9 | 1 | 1 | 3 | 5 |  |  |  |  |  |  |  |  |  |  |
| Home | 1 | 1 | 1 | 1 | 1 | 1 | 1 | 1 |  |  |  |  |  |  |  |  |  |  |
| *Route of administration:* | | | | | | | | |  |  |  |  |  |  |  |  |  |  |
| SCIT | 9 | 9 | 4 | 5,5 | 8,5 | 1 | 5 | 8 |  |  |  |  |  |  |  |  |  |  |
| SLIT | 9 | 5 | 2 | 7 | 9 | 1 | 3 | 1,5 |  |  |  |  |  |  |  |  |  |  |
| Mixtures of polymerized extracts | | | | | | | | |  |  |  |  |  |  |  |  |  |  |
| *AIT build-up dose phase:* | | | | | | | | |  |  |  |  |  |  |  |  |  |  |
| Standard | 1,5 | 1 | 1,5 | 1 | 1 | 3 | 1 | 1 |  |  |  |  |  |  |  |  |  |  |
| Cluster | 5 | 3 | 4,5 | 6 | 3,5 | 3,5 | 9 | 2,5 |  |  |  |  |  |  |  |  |  |  |
| Rush/ultrarush | 8,5 | 9 | 9 | 9 | 9 | 8 | 8,5 | 8,5 |  |  |  |  |  |  |  |  |  |  |
| *AIT schedule* | | | | | | | | |  | | | | *AIT schedule* | | | | | |
| Perennial | 7 | 9 | 9 | 5 | 9 | 8 | 9 | 8 |  |  |  |  |  |  |  |  |  |  |
| Pre-seasonal | 5 | 5 | 4,5 | 1 | 5 | 5 | 4 | 8 |  |  |  |  |  |  |  |  |  |  |
| Pre-/co-seasonal | 5 | 6 | 1,5 | 9 | 5 | 4,5 | 3 | 8 |  |  |  |  |  |  |  |  |  |  |
| *Center of administration:* | | | | | | | | |  | | | | *Center of administration:* | | | | | |
| Hospital or specialised consultation center | 1 | 5 | 9 | 9 | 8 | 4,5 | 5 | 7,5 |  |  |  |  |  |  |  |  |  |  |
| Primary care center | 8 | 9 | 9 | 9 | 4 | 8 | 9 | 7,5 |  |  |  |  |  |  |  |  |  |  |
| Home | 1 | 1 | 1 | 1 | 0,5 | 1 | 1 | 1 |  |  |  |  |  |  |  |  |  |  |
| *Route of administration:* | | | | | | | | |  | | | | *Route of administration:* | | | | | |
| SCIT | 8,5 | 9 | 9 | 9 | 9 | 9 | 9 | 9 |  |  |  |  |  |  |  |  |  |  |
| SLIT | 9 | 1 | 2 | 1 | 9 | 1 | 1 | 1 |  |  |  |  |  |  |  |  |  |  |
| **Criteria to evaluate treatment response in polyallergic patients**  *In the treatment of polyallergy, please rate from 1 to 9 the extent to which the following criteria should be considered to evaluate treatment response in polyallergic patients (where 1 = “never” and 9 =“always”).* | | | | | | | | |  |  |  |  |  |  |  |  |  |  |
| Symptom improvement (nasal and/or conjunctival) | 9 | 9 | 9 | 9 | 9 | 9 | 9 | 9 |  |  |  |  |  |  |  |  |  |  |
| Reduction in the use of medication to treat symptomatology during the acute phase | 9 | 8,5 | 9 | 9 | 7,5 | 8,5 | 9 | 9 |  |  |  |  |  |  |  |  |  |  |
| Improvement in the combined symptom and medication score (cSMS) | 9 | 8,5 | 9 | 9 | 9 | 9 | 9 | 9 |  |  |  |  |  |  |  |  |  |  |
| Improved patient-reported quality of life | 9 | 8,5 | 8 | 8 | 9 | 9 | 9 | 8,5 |  |  |  |  |  |  |  |  |  |  |
| Negative skin tests | 1 | 1 | 1 | 1 | 1 | 1 | 1 | 1 |  |  |  |  |  |  |  |  |  |  |
| Reduction of specific IgE | 1 | 2 | 1 | 1 | 2 | 1 | 2,5 | 1 |  |  |  |  |  |  |  |  |  |  |
