# Supplementary figures and images for: The Spanish REAL project: expert allergist guidance on allergen immunotherapy based on mixtures from different allergenic sources
Source: Front Allergy. 2026 Jan 30;7:1736462. doi: 10.3389/falgy.2026.1736462 (PMC12900685; doi:10.3389/falgy.2026.1736462)

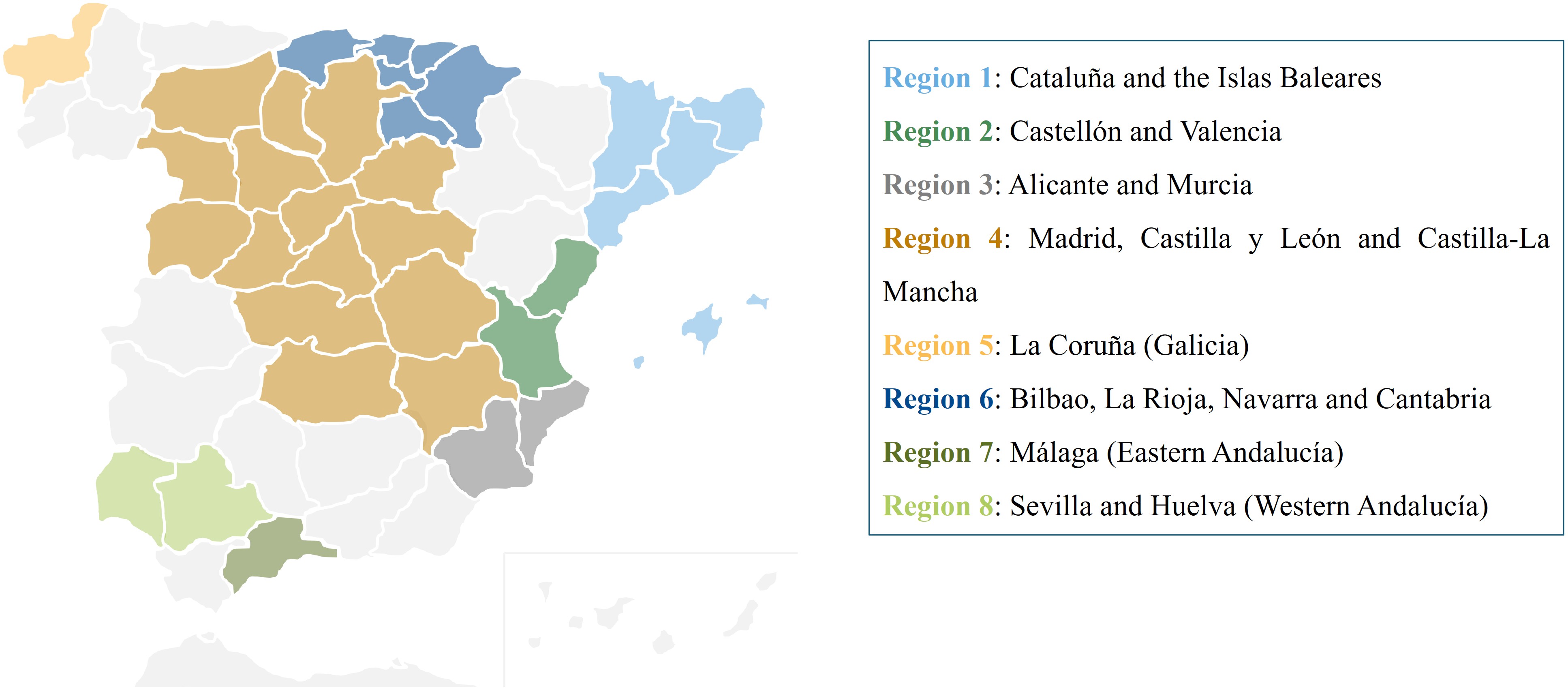

Supplement: Supplementary file 2 [file Image1.jpg]

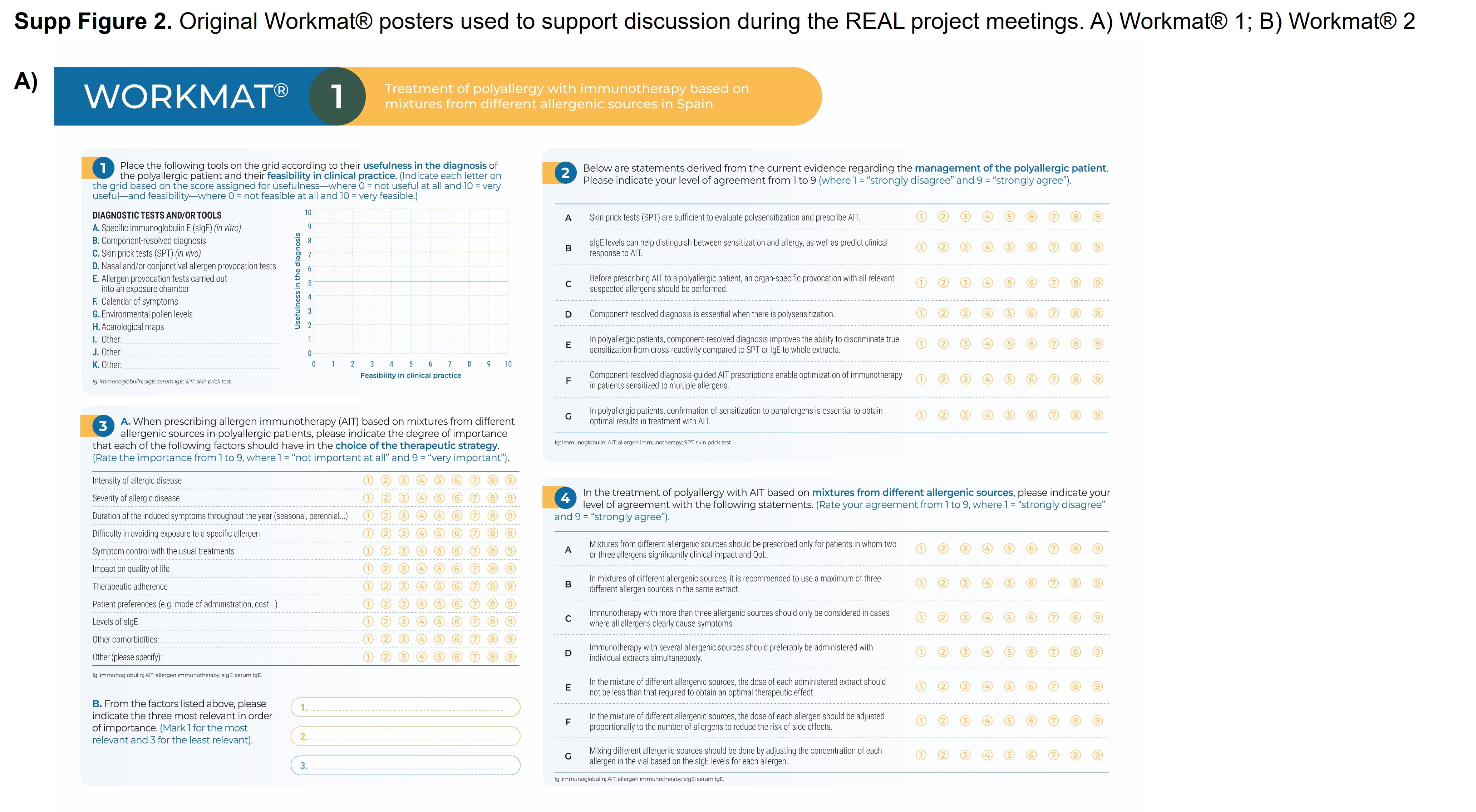

Supplement: Supplementary file 3 [file Image2.jpeg]

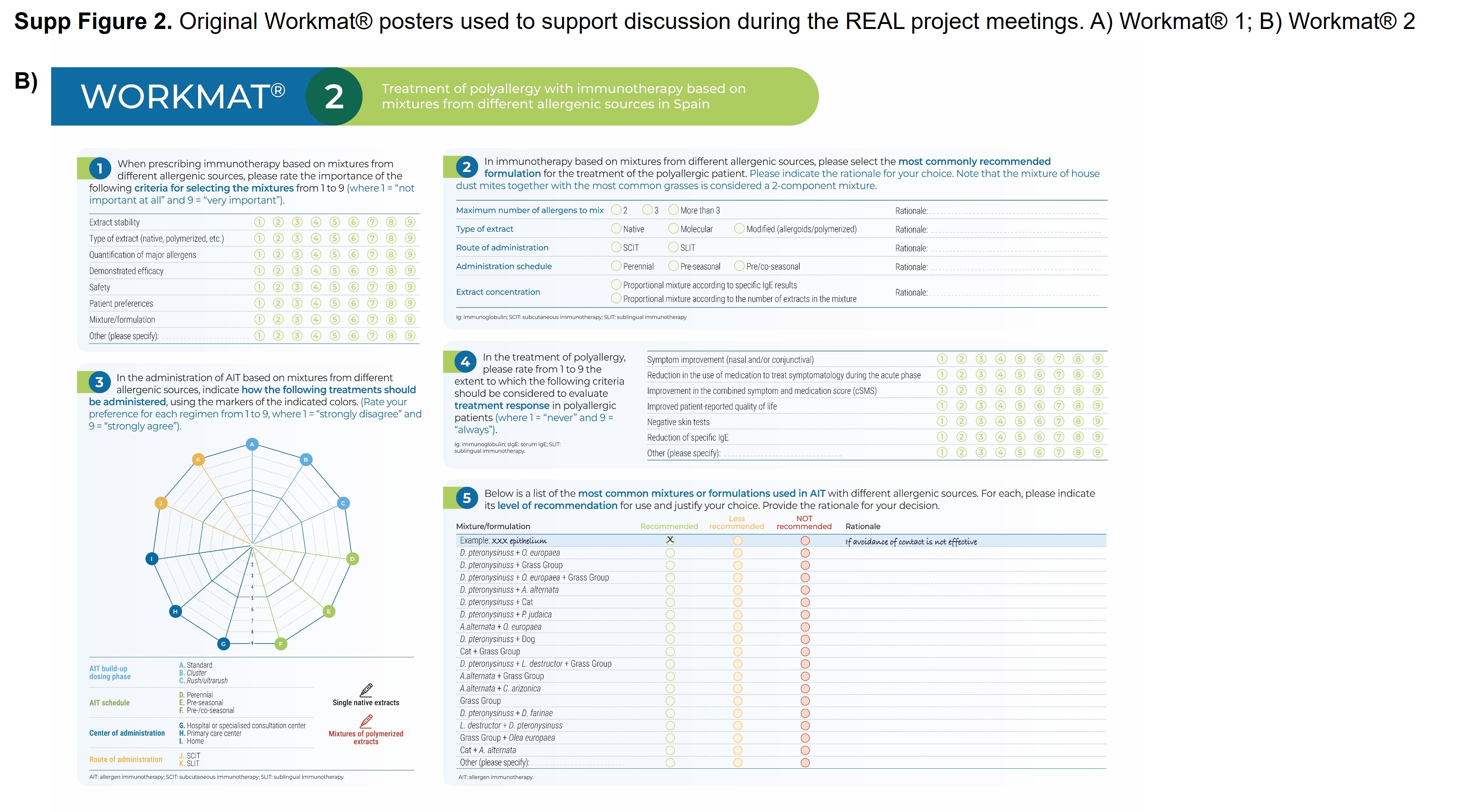

Supplement: Supplementary file 4 [file Image3.jpeg]
